# Induction of Prophages by Fluoroquinolones in *Streptococcus pneumoniae*: Implications for Emergence of Resistance in Genetically-Related Clones

**DOI:** 10.1371/journal.pone.0094358

**Published:** 2014-04-09

**Authors:** Elena López, Arnau Domenech, María-José Ferrándiz, Maria João Frias, Carmen Ardanuy, Mario Ramirez, Ernesto García, Josefina Liñares, Adela G. de la Campa

**Affiliations:** 1 Centro Nacional de Microbiología, ISCIII (Instituto de Salud Carlos III), Majadahonda, Madrid, Spain; 2 Ciber de Enfermedades Respiratorias, ISCIII, Madrid, Spain; 3 Microbiology Department, Hospital Universitari de Bellvitge-IDIBELL- Barcelona University, Barcelona, Spain; 4 Instituto de Microbiologia, Instituto de Medicina Molecular, Faculdade de Medicina, Universidade de Lisboa, Lisboa, Portugal; 5 Centro de Investigaciones Biológicas, CSIC (Consejo Superior de Investigaciones Científicas), Madrid, Spain; 6 Presidencia, CSIC, Madrid, Spain; Centers for Disease Control & Prevention, United States of America

## Abstract

Antibiotic resistance in *Streptococcus pneumoniae* has increased worldwide by the spread of a few clones. Fluoroquinolone resistance occurs mainly by alteration of their intracellular targets, the type II DNA topoisomerases, which is acquired either by point mutation or by recombination. Increase in fluoroquinolone-resistance may depend on the balance between antibiotic consumption and the cost that resistance imposes to bacterial fitness. In addition, pneumococcal prophages could play an important role. Prophage induction by fluoroquinolones was confirmed in 4 clinical isolates by using Southern blot hybridization. Clinical isolates (105 fluoroquinolone-resistant and 160 fluoroquinolone-susceptible) were tested for lysogeny by using a PCR assay and functional prophage carriage was studied by mitomycin C induction. Fluoroquinolone-resistant strains harbored fewer inducible prophages (17/43) than fluoroquinolone-susceptible strains (49/70) (*P* = 0.0018). In addition, isolates of clones associated with fluoroquinolone resistance [CC156 (3/25); CC63 (2/20), and CC81 (1/19)], had lower frequency of functional prophages than isolates of clones with low incidence of fluoroquinolone resistance [CC30 (4/21), CC230 (5/20), CC62 (9/21), and CC180 (21/30)]. Likewise, persistent strains from patients with chronic respiratory diseases subjected to fluoroquinolone treatment had a low frequency of inducible prophages (1/11). Development of ciprofloxacin resistance was tested with two isogenic strains, one lysogenic and the other non-lysogenic: emergence of resistance was only observed in the non-lysogenic strain. These results are compatible with the lysis of lysogenic isolates receiving fluoroquinolones before the development of resistance and explain the inverse relation between presence of inducible prophages and fluoroquinolone-resistance.

## Introduction

*Streptococcus pneumoniae* (the pneumococcus) is a major etiological agent of community-acquired pneumonia, meningitis and acute otitis media, as well as an important cause of acute exacerbations in patients with chronic respiratory diseases [1]. Antimicrobial resistance in the pneumococcus (including resistance to β-lactams, macrolides, tetracycline and co-trimoxazole) has expanded worldwide [2], influenced by patterns of antibiotic use and spread of a few international clones [3]. Therefore, fluoroquinolones (Fqs) are nowadays widely used for treating community-acquired pneumonia and other respiratory diseases in adults [4]. In Spain, the current prevalence of Fq resistance in pneumococci is lower than 3%, although it reaches 6.6% among strains isolated from acute exacerbations of chronic obstructive pulmonary disease [5], [6]. We have found that CC156, CC63, and CC81 are the main Fq^R^ clones since 2002 in Spain [5], [7].

Resistance to Fqs in pneumococci occurs mainly by alteration of their intracellular drug targets, i.e., DNA topoisomerase IV and DNA gyrase. Fqs inhibit these enzymes by forming a ternary complex of drug, enzyme, and DNA. Their killing effect has been related to the resolution of reaction intermediates of DNA-Fq-topoisomerase, which yield to the formation of irreparable double-stranded DNA breaks [8]. However, it has been also described that hydroxyl radical formation utilizing internal iron and the Fenton reaction are generated following gyrase poisoning and play an important role in cell killing by Fqs [9]. Fq resistance is acquired by point mutation as well as by intraspecific or interspecific recombination with streptococci of the mitis group [10]–[12]. A future increase in Fq resistance in *S. pneumoniae* would depend on the balance between antibiotic consumption and the cost that resistance imposes to bacterial fitness. A direct relationship between Fq consumption and increase in the prevalence of resistance in *S. pneumoniae* was reported [13]. We and others have reported that specific Fq-resistant (Fq^R^) mutations confer a fitness cost to *S. pneumoniae* [14], [15]. However, compensation of this fitness cost in isolates carrying recombinant topoisomerase genes has been observed [16]. In this context, if Fqs are able to induce pneumococcal prophages, they might have an important role in the emergence of Fq resistance in *S. pneumoniae* and would also modulate bacterial fitness in the presence of Fqs. Induction of prophages by Fqs has not been yet investigated in *S. pneumoniae*. This process has been described, among Gram-positive bacteria, only in *Streptococcus canis* [17], *Staphylococcus aureus* [18], [19], *Enterococcus faecalis* [20], and *Clostridium difficile* [21]. Two methods have been used to estimate rates of prophage carriage in *S. pneumoniae*, which reach different values: a) 42% was deduced from induction of bacterial lysis with mitomycin C (MitC) [22], and b) 76% was proposed from hybridization with a *lytA* bacterial probe [23]. Recently, using a PCR protocol, pneumococcal prophages have been classified into three types [24]. A study performed in 240 isolates of the CC81 clone showed multiple recombination events at the prophage region [25], suggesting that the presence of phages genes does not always equate to the presence of a functional phage.

In this study we have performed experiments of phage induction in the presence of Fqs and investigated the relation between presence of inducible prophages and Fq resistance in clinical isolates of *S. pneumoniae*.

## Materials and Methods

### Ethics statement

This study and publication of the results were approved by the “Comité Ètic d'Investigació Clínica del Hospital Universitari de Bellvitge” and the written or oral informed consent was considered not necessary, because the source of bacterial isolates was anonymized and the study was retrospective.

### Bacterial isolates

Fq^R^ [ciprofloxacin (CPX) MICs ≥4 mg/L] strains were isolated during the 2002–2009 period from 112 hospitals nationwide and previously published [5], [7]. A randomized selection of Fq^S^ isolates collected at Bellvitge Hospital during the same period was used as a control. Most isolates were from invasive sites (blood [90], cerebrospinal fluid [9], pleural fluid [18], synovial fluid [2]), and respiratory tract samples (sputum [125], bronchoalveolar lavage [12]). Clonal complex (CC) characterization was made on the basis of pulsed-field gel electrophoresis (PFGE) and assessed by multilocus sequence typing (MLST). Briefly, genomic DNA embedded in agarose plugs was restricted with SmaI or ApaI and fragments were separated by PFGE in a CHEF-DRIII apparatus (Bio-Rad). PFGE patterns were compared with representative clones of the Pneumococcal Molecular Epidemiology Network (PMEN), the world-wide epidemic clones [3]. Isolates with patterns varying by three or less bands were considered to represent the same PFGE type. In order to assess the identity with global pneumococcal clones, at least one isolate of each PFGE pattern/serotype combination was analyzed by MLST. Allele numbers and sequence types (ST) were assigned using the MLST web site (http://www.mlst.net).

### Detection of phage DNA

PCR detection of the *hol1* gene, indicative of the presence of phage, was performed as described previously [24]. Strains with *hol1* positive PCR were tested for the presence of *int1*, *int2* and *mtp* with specific oligonucleotide pairs to identify phage types. Fragments of *parC* or *rpoB* genes, which were amplified with oligonucleotides parC50/parC152, or rpoB428/rpoB474R [14], respectively, were used as controls. Strains 949, which carry the type 1 prophage MM1, and R6 (no prophage), were used as controls. For phage induction, cultures were grown exponentially in Todd-Hewitt medium supplemented with 0.5% of yeast extract (THY) at 37°C until OD_620_ = 0.1. Then, growth kinetics of isolates with and without the addition of 75 ng/mL MitC was monitored by OD_620_ measured every 15 min during a 4-h period. The induction with MitC was considered positive when a 2-to 3-fold decrease in OD with respect to the untreated culture was observed after 2-to 3 h of treatment.

### Southern blot hybridizations

Cultures were grown exponentially until OD_620_ = 0.1. At this time (time 0) antibiotics were added at concentrations equivalent to 1×MIC for each strain and samples were taken during 4 h. Phage and chromosomal DNAs were obtained as follows. First, 2.92 g of NaCl were added to 50 mL of culture, and the solution kept at 4°C for 1 h. The suspension was centrifuged at 2,200×*g* for 20 min at 4°C. The supernatant, containing phages, was precipitated overnight at 4°C with polyethylene glycol 800 (1 g/10 mL solution). The suspension was centrifuged at 11,200×*g* during 15 min at 4°C and the pellet suspended in 0.5 mL of buffer (100 mM Tris-HCl pH 7.5, 0.1 M NaCl, 10 mM MgCl_2_). To eliminate cellular nucleic acids, the phage suspension was treated with RNase and DNase (100 μg/mL each) during 1 h at 37°C. Phage particles were digested 90 min with proteinase K (150 μg/mL) in the presence of 0.5% SDS and 50 mM EDTA at 50°C. Phenol extraction and ethanol precipitation allowed the isolation of phage DNA. A 191-bp phage probe containing *hol1* was obtained with biotinylated P9 and P9-R oligonucleotides [24] using strain 949 as template. The Phototope-Star Detection Kit (New England Biolabs) was used following the manufacturer's instructions.

### Statistical analysis

The Fisher exact test (χ^2^ test) was used when appropriate. *P*<0.05 was considered significant.

## Results

### Induction of prophages by Fq treatment

**Figure 1:**
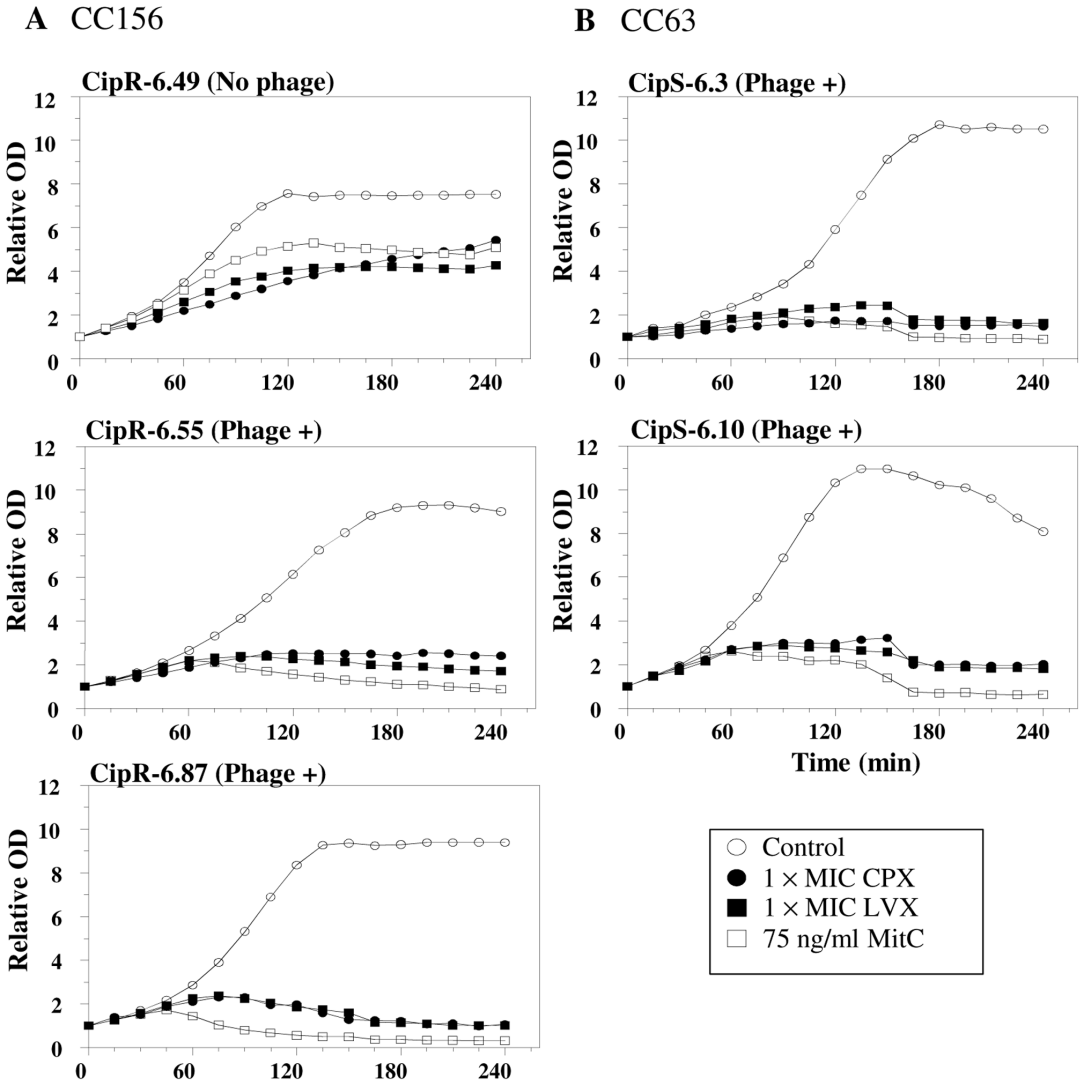
Lysogenic isolates exhibited lysis in the presence of mitomycin C and fluoroquinolones. The growth kinetics of three isolates of the CC156 clone (one non-lysogen and two lysogens) and two lysogenic isolates of the CC63 clone were followed in the presence of fluoroquinolones or MitC. Cultures growing exponentially in THY at 37°C to OD_620_ = 0.1 were divided and treated as indicated. Growth was monitored every 15 min during a 4-h period.

**Figure 2:**
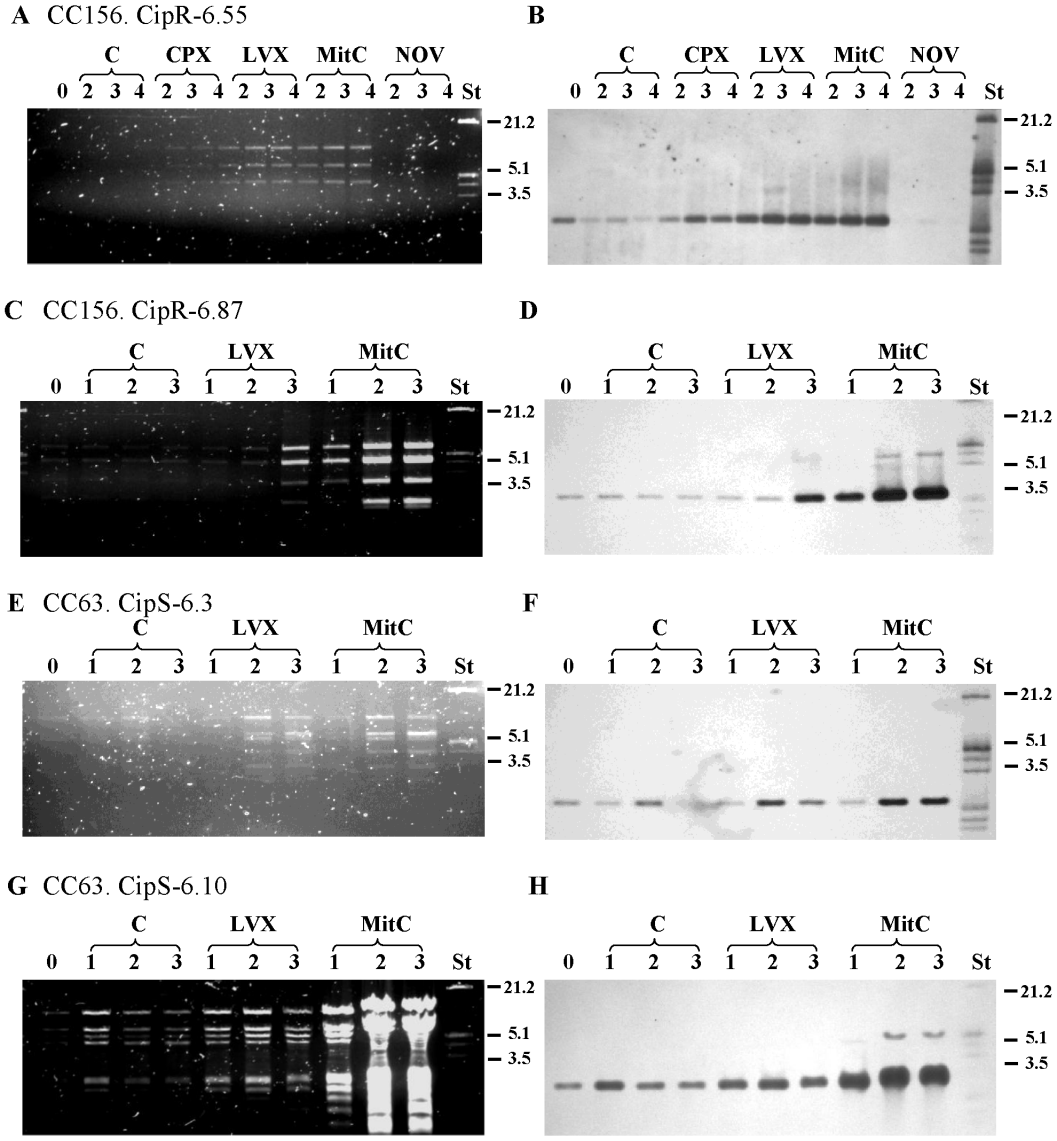
Prophage induction with Fqs and mitomycin C was detected by Southern-blot hybridization. Cultures were grown either without treatment (C), or in the presence of antibiotics at concentrations equivalent to 1×MIC: 32 μg/ml of ciprofloxacin (CPX); 1–16 μg/ml of levofloxacin (LVX); 1 μg/ml of novobiocin (NOV). MitC was used at 75 ng/ml. Phages were concentrated from the supernatant as described in methods at the beginning of the experiment (time 0) and at 1-to 4 h after treatment. (A, C, E. and G) Amounts of DNA equivalent to 0.5 OD_620_ units of the culture were digested with EcoRV, run in 0.8% agarose gels, and stained with ethidium bromide. (B, D, F and H) The DNA bands were blotted to a nylon membrane and hybridized with a *hol1*-probe.

First, the kinetics of growth of two isolates, (CipR-6.55 and CipR-6.49) belonging to the CC156 clone was analyzed in the presence of Fqs and MitC, a typical prophage inducer. Treatment with CPX and levofloxacin (LVX) inhibited the growth of both isolates in a concentration-dependent manner, being the inhibition more evident in CipR-6.55 (carrying phages of types 1 and 2) than in CipR-6.49 (no prophages), which suggested prophage induction by the drug. In accordance, CipR-6.55 showed lysis after treatment with MitC (Figure 1). This lysis was also observed in three additional isolates, one of CC156 (CipR-6.87, with two phages of types 1 and 2), and two of CC63 with phages of type 2 (CipS-6.3 and CipS-6.10). Phage induction was also followed by Southern blotting. First, phage induction was tested in CipR-6.55 in the presence of CPX and LVX, MitC and the gyrase B inhibitor novobiocin. Phage DNA was purified, digested with EcoRV and hybridized with a *hol1* probe. As shown in Figure 2, discrete restriction bands were detected in the culture supernatants, which corresponded to phage DNA. Some basal spontaneous induction of phage was also observed in the untreated cultures. However, the amount of phage DNA, as detected by hybridization, was higher when Fqs or MitC were used. These results showed induction of prophages by both Fqs and MitC, but not by novobiocin. Given that induction by LVX was more efficient than by CPX, the first Fq was chosen for further studies. In three additional lysogenic isolates studied, induction with LVX and MitC was also observed (Figure 2).

### Detection of prophages by PCR in a collection of S. pneumoniae isolates

A total of 265 clinical isolates were tested for the presence of phages [105 Fq^R^ and 160 Fq-susceptible (Fq^S^)]. Of them, 113 (42.6%) carried prophage DNA, and no significant difference was found among Fq^R^ (40.9%; 43/105) and Fq^S^ (43.5%; 70/160). Likewise, no difference was observed among prophage carriage rates of isolates collected from invasive samples (36.9%; 45/122) and respiratory tract samples (46.7%; 64/137). Types 1 and 2 prophages were more abundant than those of type 3. A majority (77/113) of isolates carried prophages of a single type, (51 isolates with type 1 phages, 25 isolates with type 2 phages and 1 isolate with a type 3 phage), 16 isolates carried prophages of two types (11 isolates with types 1 and 2, and 5 isolates with types 2 and 3), and 6 isolates carried prophages of the three types. Fourteen isolates showed amplification with *hol1* oligonucleotides but not with those specific for *int1*, *int2* or *mtp* suggesting the presence of prophage remnants or of temperate phages of an unknown type.

### Presence of inducible prophages among isolates of prevalent clones

Since we showed a correlation between prophage induction by MitC and Fqs (Figures 1 and 2), MitC induction was chosen to test the functionality of the prophages detected by PCR in all lysogenic isolates. The kinetics of growth in the presence of MitC was analyzed as described in methods. Among 113 lysogenic isolates, only 66 (58.4%) exhibited detectable lysis after treatment with MitC. The frequencies of functional prophages were statistically lower (*P* = 0.0018) in Fq^R^ (17/43) than in Fq^S^ (49/70) isolates (Table 1). No difference was observed in the distribution of functional phages among invasive (28/122) and respiratory tract samples (37/137).

**Table 1:** Relevant characteristics of *S. pneumoniae* isolates and their prophages analyzed in this study.

				Type of phage						
CC^a^ (no. of isolates)	Phenotype^b^	*hol1* +^c^	MitC +^d^	1	2	3	1+2	1+2+3	2+3	Other
CC180 (30)	29 S	22	21	20	−	−	−	−	−	2
	1 R	0	0	−	−	−	−	−	−	−
CC306 (29)	28 S	1	1	−	−	−	−	−	−	1
	1 R	0	0	−	−	−	−	−	−	−
**CC156 (25)**	6 S	1	1	−	1	−	−	−	−	−
	19 R	3	2	−	1	−	2	−	−	−
CC30 (21)	17 S	8	4	2	2	−	−	−	−	4
	4 R	1	0	−	−	−	−	1	−	−
CC62 (21)	19 S	14	8	6	4		2	−	−	2
	2 R	2	1	2	−	−	−	−	−	−
**CC63 (20)**	7 S	2	2	−	−	−	−	−	−	−
	13 R	1	0	−	−	−	−	−	−	1
CC230 (20)	18 S	6	4	1	3	−	−	−	−	2
	2 R	1	1	1	−	−	−	−	−	−
**CC81 (19)**	7 S	2	0	−	−	2	−	−	−	−
	12 R	4	1	−	3	−	−	−	−	1
CC97 (6)	3 S	3	1	3	−	−	−	−	−	−
	3 R	3	0	2	−	−	−	−	−	1
CC433 (6)	3 S	1	1	1	−	−	−	−	−	−
	3 R	2	0	1	−	−	1	−	−	−
CC42 (5)	4 S	0	0	−	−	−	−	−	−	−
	1 R	0	0	−	−	−	−	−	−	−
CC717 (5)	3 S	3	1	3	−	−	−	−	−	−
	2 R	2	2	2	−	−	−	−	−	−
CC17 (4)	2 S	2	2	−	2	−	−	−	−	−
	2 R	2	1	−	2	−	−	−	−	−
CC90 (4)	2 S	2	1	−	1	−	−	1	−	−
	2 R	2	1	−	1	−	−	1	−	−
CC260 (4)	2 S	0	0	−	−	−	−	−	−	−
	2 R	1	0	1	−	−	−	−	−	−
CC67 (3)	2 S	2	2	2	−	−	−	−	−	−
	1 R	0	0	−	−	−	−	−	−	−
CC191(3)	2 S	0	0	−	−	−	−	−	−	−
	1 R	0	0	−	−	−	−	−	−	−
CC88 (2)	2 S	0	0	−	−	−	−	−	−	−
	0 R	0	0	−	−	−	−	−	−	−
CC247 (2)	2 S	0	0	−	−	−	−	−	−	−
	0 R	0	0	−	−	−	−	−	−	−
CC989 (2)	1 S	0	0	−	−	−	−	−	−	−
	1 R	0	0	−	−	−	−	−	−	−
Others (34)	1 S	1	0	−	1	−	−	−	−	−
	33 R	19	8	4	3	1	3	−	3	5

a Clones are named by their clonal complex number. Those showed in boldface and underlined are the main clones involved in Fq resistance in Spain since 2002.

b Isolates are separated on the basis of their Fq susceptibility: S, susceptible (CPX MICs ≤2 mg/L); R, resistant (MICs ≥4 mg/L).

c PCR detection for *hol1* gene.

d Functional phages caused cell lysis in the presence of MitC.

As shown in Table 1, several clones had low presence of phage DNA: CC156; CC81; CC63; and CC306. No association between the type of phage carried and the genotype was observed. Isolates of the same clone carried different phage types or combinations, with the exception of CC180 in which 20 out of 22 isolates carried a type 1 prophage. Four clones: CCT156 (3/25); CC63 (2/20); CCT81 (1/19) and CC306 (1/29) showed low rates of functional prophage carriage. The first three are the main Fq^R^ Spanish clones since 2002 [5], [7] whereas, CC306, is usually antimicrobial susceptible. On the other hand, isolates belonging to CC180 (21/30); CC30 (4/21); CC230 (5/20); and CC62 (9/21) showed higher frequencies of functional prophage carriage. These last four clones were low prevalent among Fq^R^ Spanish pneumococci [5], [7].

### Low frequency of inducible prophages among persistent strains isolated from patients that received Fq treatment

To test the role of Fq therapy in phage induction *in vivo*, 11 persistent pneumococci collected from 10 adult patients with chronic respiratory diseases were studied (Table 2). Details of these strains have been previously reported [26]. All isolates from each patient were clonally related (same MLST) and were repeatedly isolated throughout the period (27 to 165 weeks) in which these patients received multiple Fq therapy courses. Only isolates of two patients (7 and 11) showed a positive detection of the *hol1* gene. Moreover, only prophages from the pneumococci of patient 7 were induced with MitC. Four strains were Fq^R^ since the first isolation and did not carry any inducible prophages, although one of them had a phage remnant (patient 11). Among the five Fq^S^ strains that did not carry prophages, two developed resistance after Fq course (patients 7 and 9). Patient 7 was sequentially colonized by two different strains. Although the first one (of ST63^15A^) carried functional prophage and developed Fq resistance, it was replaced by a new nonlysogenic strain (ST558^35B^) after Fq therapy [26].

**Table 2:** Persistent *S. pneumoniae* strains causing ≥3 episodes of acute exacerbations in patients with chronic respiratory diseases.

Patient ID	Clone	Episode	Fq phenotype^a^	*hol1*/MitC b
1	ST156^9V^	1^st^ to 3^rd^	LL-R	−
3	ST838^9V^	1^st^ to 4^rd^	S	−
4	ST838^9V^	1^st^ to 3^rd^	HL-R	−
5	ST838^9V^	1^st^	HL-R	−
		2^nd^	LL-R	−
		3rd	HL-R	−
7	ST63^15A^	1^st^, 2^nd^	S	+/+
		3^rd^, 4^rd^, 6^th^	HL-R	+/+
	ST558^35B^	5^rd^, 7^th^, 8^th^	S	−
8	ST63^15A^	1^st^	S	−
		2^nd^ to 5^th^	LL-R	−
9	ST88^19F^	1st	S	−
		2^nd^ and 3^rd^	HL-R	−
10	ST87^19F^	1^st^ to 5^th^	S	−
11	ST2100^19F^	1^st^ to 3^rd^	HL-R	+/−
12	ST276^19A^	1^st^ to 3^rd^	S	−

a S, susceptible (CPX MICs ≤2 mg/L); LL-R, low- level of resistance (MICs 4–8 mg/L); HL-R, high level of resistance (MICs ≥16 mg/L).

b *hol1* + indicates PCR detection for *hol1* gene; MitC + indicates cell lysis of the culture in the presence of MitC.

### The presence of a prophage affects development of CPX resistance

**Figure 3:**
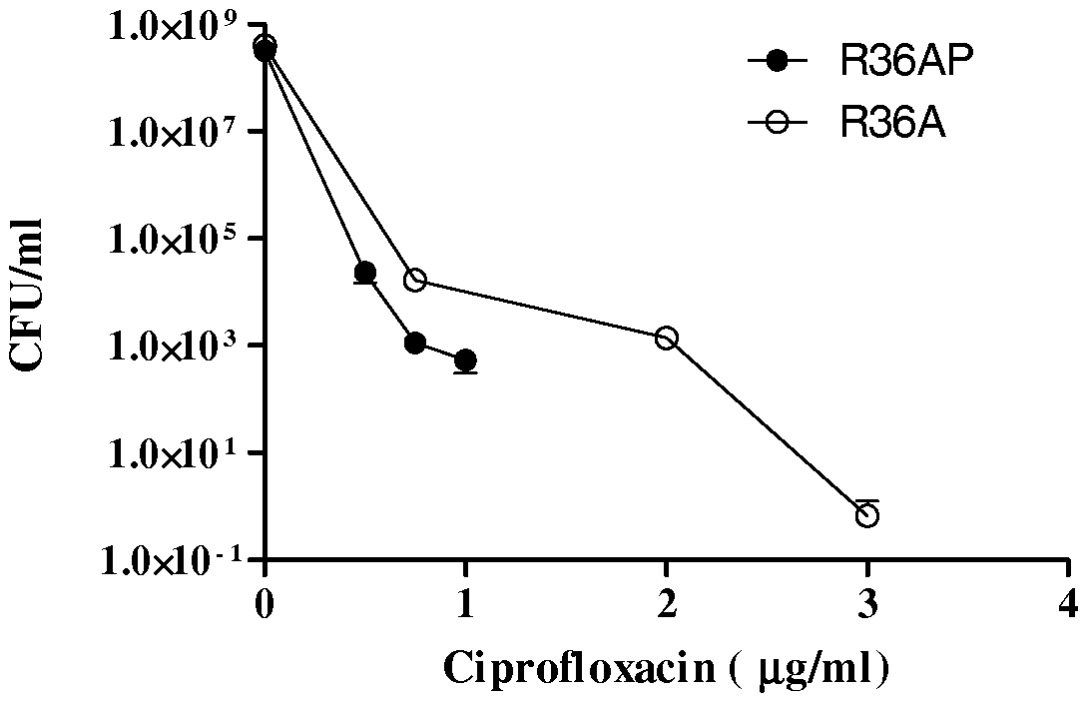
Non-lysogenic R36A strain developed CX-resistance while lysogenic R36A (R36AP) did not. Overnight cultures of R36A and R36AP were grown in THY until OD_620_ = 0.4, then these were diluted 20-fold in 500 ml of the same medium and grown until OD_620_ = 0.1. At this point CPX was added to the cultures to reach 1×MIC (0.5 μg/ml). Cultures were grown for 4 h and bacteria were recovered by centrifugation at 5000 g for 30 minutes. Cells were suspended in THY +25% glycerol at concentrations of 3.3×10^8^ and 4.1×10^8^ CFUs/ml for R36A and R36AP, respectively. Bacteria were plated in THY agar plates containing the indicated CPX concentrations. Results are the means ±SD of three independent replicates.

The observation that Fq^R^ isolates have lower rates of inducible prophages than Fq^S^ pneumococci, together with the results of induction of phages by Fq^s^, suggest that under Fq selective pressure, lysogenic pneumococci will be prone to die due to phage-mediated lysis, while non-lysogenic isolates would be able to develop Fq resistance. Resistance would be hence more likely to arise in isolates that do not carry prophage. To test this hypothesis, two isogenic strains, R36A (wild type) and R36AP (an R36A derivative carrying an inducible prophage) [27] were cultured for 4 h in the presence of 1×MIC of CPX (0.5 μg/ml) and the presence of quinolone resistant mutants was evaluated by plating on several CPX concentrations (Figure 3). We assumed that phage-mediated lysis would occur preferentially in liquid medium; although we cannot discard that lysis is also occurring on plates. At 4×MIC of CPX (2 μg/ml), there were no colonies in the R36AP strain, while a total of 1.4×10^3^±0.2×10^3^ (mean ± SD) colonies (frequency of 3.5×10^−5^) were obtained from the R36A strain. Among them, 8 isolates were selected and their *parC* QRDR regions were sequenced as described [5], [7]. Results showed that a majority (6 out of 8) carry the S79Y mutation, a classical mutation known to be involved in CPX resistance. At 3 μg/ml CPX, 2 colonies of the R36A strain were obtained (frequency of 5×10^−9^), one of these also carried the ParC S79Y change. These results are consistent with a deleterious effect of prophage carriage for the development of quinolone resistance in pneumococci.

## Discussion

In this study we first showed that Fqs targeting topoisomerase IV, such as CPX and LVX, are able to induce the lytic cycle of pneumococcal temperate phages. Comparison of growth kinetics in the presence of 1×MIC of these fluoroquinolones showed lower OD increases in isolates carrying inducible prophages, than in the non-lysogenic CipR-6.49 isolate. This behavior was related with the induction of cell lysis by the prophages. In contrast, novobiocin, an inhibitor of the DNA gyrase, was unable to induce lysis of CipR-6.55. All these results suggest a role of the inhibition of topoisomerase IV in the lysis response. This could be a consequence of the cellular processes acting on the ternary complex formed by topoisomerase IV-Fq and DNA [8]. However, it could also be due to transcriptional regulation of phage or bacterial genes by changes in DNA supercoiling caused by inhibition of topoisomerase IV, given that treatment of *S. pneumoniae* with LVX causes a complex transcriptomic response [28]. On this respect, it has been shown that the transcription of the pneumococcal *recA* gene, a competence-induced gene, is necessary for temperate phage induction [29], and that competence in *S. pneumoniae*, a bacterium lacking an SOS-like system, is induced by Fqs and MitC but not by other antibiotics [30].

Since our results showed that Fqs caused bacterial lysis by phage induction, we determined the rates of inducible phages in isolates of the most frequent clones causing pneumococcal diseases in adult patients. Using a PCR approach, we determined that about half of the isolates analyzed were lysogenic, a figure compatible with the previously reported value (42%) based on MitC induced bacterial lysis [22], but lower than the 76% estimated by detection of the prophage *lytA*-like gene by hybridization with a host *lytA* probe [23]. However, both the PCR detection and the hybridization approaches overestimate the rate of inducible, functional prophage carriage, since these methods detect also defective prophages, and experiments of induction with MitC were necessary to determine the functional phage rate.

We showed that Fq^R^ isolates have lower rates of inducible prophage carriage than Fq^S^ pneumococci. These findings, together with the induction of phages by Fqs, and the inability to select FqR isolates in the R36AP strain in conditions when these arise readily in the non-lysogenic parental strain R36A, suggest that under Fq pressure lysogenic pneumococci will be prone to die due to phage-mediated lysis, while non-lysogenic isolates are able to develop Fq resistance. Consistent with this hypothesis, isolates belonging to the three main Fq^R^ Spanish clones (CC156, CC63, and CC81), have a frequency of inducible prophages lower than clones not related with Fq resistance, such as CC30, CC62 or CC180. In contrast, the vast majority of isolates of CC306, which were Fq^S^, were non-lysogenic. There are two possible explanations for this finding. The first is that isolates of this clone usually cause invasive pneumococcal disease in children, who are not treated with Fqs. The second is that, since this clone is rarely found as a colonizer (neither in children nor adult patients with chronic obstructive pulmonary disease), it may seldom exchange DNA with other streptococci or have the chance to be infected by temperate bacteriophages.

Another possibility could be that the clones within which Fq resistance is most common are less likely to be lysogenic for unrelated reasons. However, our results shown that there are differences in the prevalence of inducible phage between different clones not commonly resistant to Fq, ranging from 1/29 to 21/30 (Table 1) and experiments with isogenic strains differing only in the carriage of a prophage support a role of prophages in preventing the development of Fq resistance.

Finally, we found a low frequency of functional prophages (1/11) in strains persistently colonizing patients which received multiple courses of Fq therapy. These results also support the role of prophage in cell lysis and development of *in vivo* Fq resistance in *S. pneumoniae*. This ecological niche is optimal for the development of antibiotic resistance; given that the patients had multiple infections with different pathogens and that they received multiple courses of antibiotic treatment. In relation with fluoroquinolone treatments, the doses of CPX that are able to kill Gram-negative bacteria are subinhibitory for *S. pneumoniae* and this would allow, both the development of resistance in this kind of patients, and also the induction of prophages.

The evolution of bacteria cannot be understood without the contribution of their prophages [31]. These could change from inducible to cryptic prophages (unable to excise from the chromosome and cause cell lysis), which contribute significantly to resistance to sub-lethal concentrations of Fqs and β-lactam antibiotics primarily through phage-encoded proteins that inhibit cell division, as recently demonstrated for *Escherichia coli* prophages that do not excise on MitC treatment [32]. Lysogeny is also important for interspecies competition, as showed by the killing of *S. aureus* by prophage induction caused by H_2_O_2_ production by *S. pneumonia*e in the nasopharynx [19]. Activation of key proteins involved in phage-induced cell lysis, encoded either by the prophages or by the bacterial host, may be a novel way to fight antimicrobial resistance.
